# Apela improves cardiac and renal function in mice with acute myocardial infarction

**DOI:** 10.1111/jcmm.15651

**Published:** 2020-07-20

**Authors:** Yang Pan, Quanyi Li, Hong Yan, Jin Huang, Zhi Wang

**Affiliations:** ^1^ Department of Cardiovascular Medicine Nanjing Brain Hospital Nanjing Medical University Nanjing China; ^2^ Department of Cardiovascular Medicine Nanjing Chest Hospital Nanjing China; ^3^ Department of Clinical Laboratory Nanjing Brain Hospital Nanjing Medical University Nanjing China; ^4^ Department of Clinical Laboratory Nanjing Chest Hospital Nanjing China

**Keywords:** apela, apelin peptide jejunum receptor, cardiac function, myocardial infarction, renal function

## Abstract

Apela was recently identified as a new ligand of the apelin peptide jejunum (APJ) receptor. The purpose of this study was to investigate the role of apela in post‐myocardial infarction (post‐MI) recovery from cardiorenal damage. A murine MI model was established, and apela was then infused subcutaneously for two weeks. Echocardiographs were performed before and after infarction at the indicated times. Renal function was evaluated by serum and urine biochemistry. Immunohistochemistry of heart and kidney tissue was performed by in situ terminal deoxynucleotidyl transferase‐mediated dUPT nick end‐labelling reaction. Compared to the control group (MI/vehicle), the average value of the left ventricular ejection fraction in apela‐treated mice increased by 32% and 39% at 2‐ and 4‐week post‐MI, respectively. The mean levels of serum blood urea nitrogen，creatinine, N‐terminal pro‐brain natriuretic peptide and 24‐hour urine protein were significantly decreased at 4‐week post‐MI in apela‐treated mice relative to that of control animals. At the cellular level, we found that apela treatment significantly reduced myocardial fibrosis and cellular apoptosis in heart and kidney tissue. These data suggest that apela improves cardiac and renal function in mice with acute MI. The peptide may be potential therapeutic agent for heart failure.

## 1 INTRODUCTION

In recent years, research has found a non‐coding RNA transcription gene called Apela (Elabela or Toddler); this gene can be transcribed and translated to produce a secreted hormone containing 55 amino acids, which becomes a mature short peptide of 32 amino acids after enzymatic cleavage. Apela and apelin can also act on an orphan G protein‐coupled receptor called apelin peptide jejunum (APJ) receptor.^1^, ^2^ Previous studies showed that APJ receptors are widely expressed in human tissues and organs.^3^ Exogenous apela can interact with APJ receptors in mammalian tissues and cells to exert corresponding biological effects.^4^, ^5^, ^6^, ^7^, ^8^, ^9^, ^10^ Previous basic experiments have also shown that apelin can improve cardiac function in mice with myocardial infarction by inhibiting myocardial cell apoptosis, promoting angiogenesis and inhibiting myocardial fibrosis.^11^ Sustained apela gene therapy can improve blood pressure in high‐salt diet hypertension rats.^12^ Exogenous apela can also reduce pulmonary arterial pressure in mice pulmonary hypertension models.^13^ Recently, clinical studies have shown that endogenous plasma apela levels are higher in patients with acute myocardial infarction (MI) than in healthy controls; furthermore, apela levels are also negatively correlated with left ventricular ejection fraction (LVEF).^14^ These basic experiments and clinical studies suggest that apela is valuable in the diagnosis and treatment of cardiovascular disease. In this study, we prepared a heart failure model of MI in mice to further investigate the effects of exogenous apela intervention on the heart and kidney function of mice with AMI as well as to explore the possible underlying mechanisms.

## 2 MATERIAL AND METHODS

### 2.1 Drug

The peptide of apela with a full length of 32‐aa (QRPVNLTMRRKLRKHNCLQRRCMPLHSRVPFP) is synthesized by Hangzhou DGpeptides Co., Ltd with a purity of >98%.

### 2.2 Preparation of MI model

Animal experiment was approved by the Committee on Ethics in the Care and Use of Laboratory Animals of Nanjing Medical University. Eight‐week‐old male SPF‐grade C57BL/6 mice were purchased from Shanghai Xipuer‐Bikai Experimental Animal Co., Ltd. Mice had ad libitum access to food and water in the breeding environment, and the indoor temperature was controlled at 23°C ± 2°C. Mice were intraperitoneally injected with 10% chloral hydrate (0.02 mL/10 g), and the trachea was intubated with a ventilator to control ventilation frequency: 133 breaths/min, tidal volume (1–1.5 mL)]. The chest was opened to expose the heart, and then, a 7‐0 suture was used to ligate the left anterior descending coronary artery about 2 mm below the left atrial appendage. The sham‐treated group underwent a similar procedure without ligation. Echocardiography was performed at 2 and 4 weeks after model preparation as previously described.^11^ Briefly, mice were anesthetized with 2% isoflurane inhalation, and echocardiography was performed using a small animal ultrasound system (Vevo770, Canadian VISUALSONICS company) equipped with a 30‐MHz probe. M‐mode echocardiography was used to measure the left ventricular end‐systolic and end‐diastolic inner diameters, and the LVEF was calculated automatically.

### 2.3 Grouping and drug administration

The experimental animals were divided three groups: sham operation procedure with saline treatment (sham + vehicle), MI with apela treatment (MI + apela), and MI with saline treatment (MI + vehicle). Apela (1 mg/kg/d) or an equal volume of saline was continuously administered for 2 weeks by a micro‐dosing osmotic pump (Alzet Model 1002, Alza Corp, Mountain View, CA, USA) implanted subcutaneously in the abdomen of mice.

### 2.4 Blood and urine biochemistry

Serum endogenous apela (Eiaab, Wuhan, China) and NT‐proBNP (Cloud‐Clone Corp, Houston, TX, USA) was measured by ELISA according to the manufacturer’s instructions. The serum urea nitrogen (BUN) and creatinine (Cr) were respectively detected using ELISA kits (Creative Diagnostics, USA) according to user’s manual. The 24‐h urine volume of mice was respectively collected before operation and 2 and 4 weeks after operation. The 24‐h urine protein content was measured with a urine protein quantitative test box (Jiancheng bioengineering, Nanjing, China).

### 2.5 Cardiac 2, 3, 5‐triphenyltetrazolium chloride staining

Mice were euthanized by intraperitoneal injection of excessive pentobarbital. The heart was quickly removed and was rinsed with ice‐cold saline to remove blood stains; it was then immediately frozen at −80℃ in a refrigerator for 3 min. Sections of the ventricle tissue were cut at 2‐mm thickness and placed in 1% 2, 3, 5‐triphenyltetrazolium chloride solution (Sangon Biotech, Shanghai, China); this was followed by incubation at 37°C in the dark for 30 min. The tissues were removed from the solution and photographed in sequence. The infarct area was measured using Image Pro Plus software. The infarct area (%) = the area of the infarcted area/the area of the left ventricle × 100%.

### 2.6 Heart tissue histology

Briefly, heart tissues were fixed with 4% formalin and embedded in paraffin. The 5‐μm‐thick sections were prepared and stained with Masson’s trichrome stain according to the manual of Masson’s trichromatic Staining Kit (KeyGen Biotech, Nanjing, China).

### 2.7 Immunohistochemical detection

Heart or kidney tissues were embedded in paraffin before staining. Then, 5‐μm‐thick sections were cut, deparaffinised and hydrated. After antigens were retrieved, endogenous peroxidase was blocked by incubation in 3% hydrogen peroxide. The tissue sections were incubated overnight at 4℃ with primary antibody anti‐CD31 (ab28364, Abcam, Cambridge, UK, 1:50) or MPO (ab208670, Abcam, Cambridge, UK, 1:1000). Samples were then incubated with a biotinylated secondary antibody and sequentially incubated in ABC‐peroxidase solution. Finally, samples were stained with 3, 3’‐diaminobenzidine, slightly counterstained with haematoxylin and fixed with neutral balata.

### 2.8 TUNEL assay

Sections were detected by TUNEL assay using an In Situ Cell Death Detection Kit (KGA703, KeyGen Biotech, Nanjing, China). Briefly, heart or kidney tissue sections with paraffin‐embedded were deparaffinised, rehydrated and antigen‐retrieved. The following procedures were according to the instructions of the TUNEL assay kit.

### 2.9 Statistical analysis

GraphPad Prism5 statistical software was used for data analysis. The data were expressed as mean value ± SD. The statistical differences were assessed using one‐way ANOVA or Student’s unpaired t test. A *P* value of <0.05 was considered significant.

## 3 RESULTS

### 3.1 Apela improves heart function in mice after MI

**FIGURE 1:**
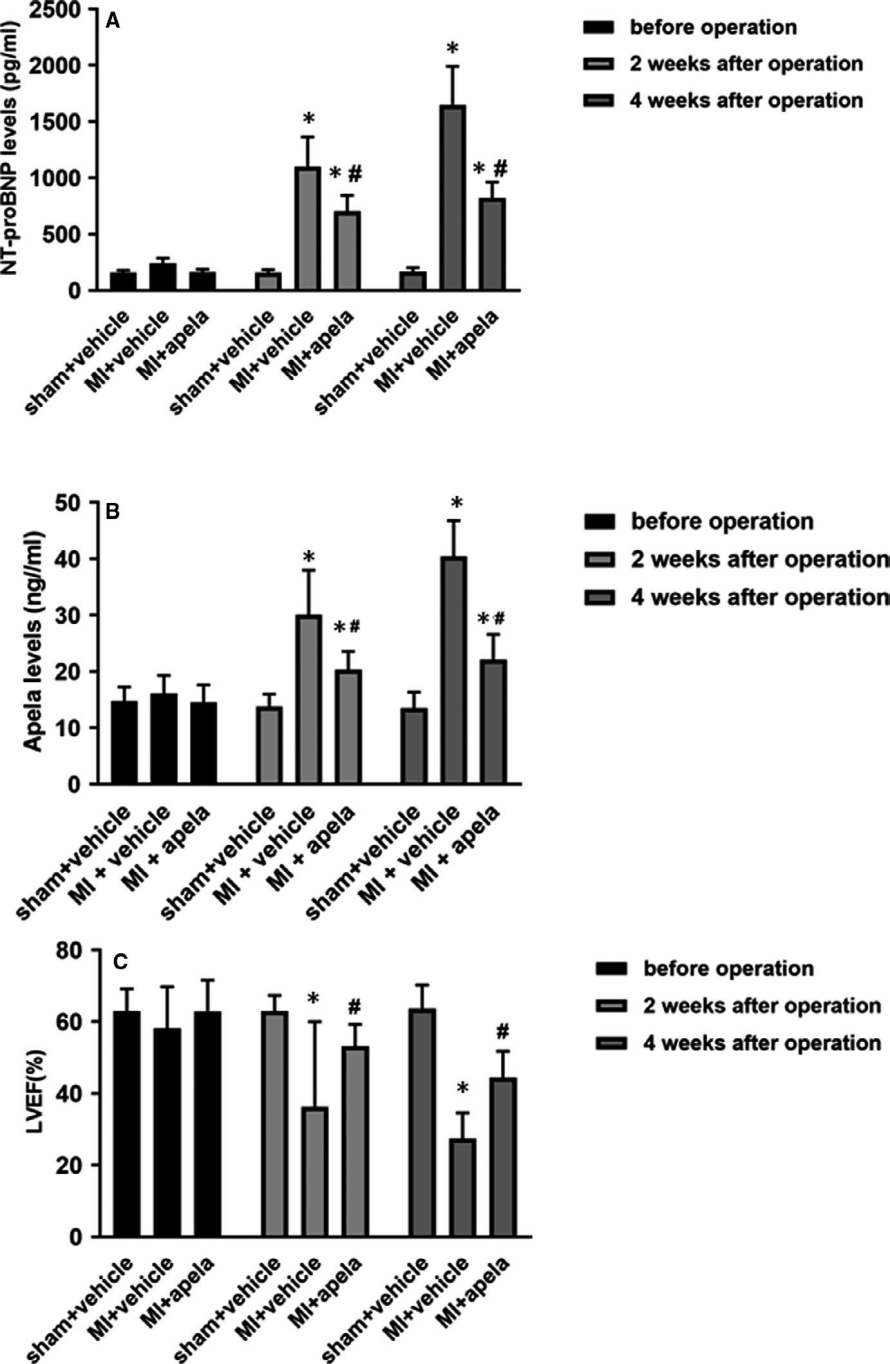
The mice cardiac function at different times. Serum levels of NT‐proBNP (A) and apela (B) in mice. The values of LVEF in mice (C). The results were presented as mean ± SD (n = 5). *P < 0.05, vs. sham + vehicle group; ^#^ *P* < 0.05, vs. MI + vehicle group

There was no significant change in serum NT‐proBNP or apela concentration in sham‐treated mice before MI and at 2 and 4 weeks after MI, whereas the NT‐proBNP or apela concentration in the control group (MI + vehicle) increased gradually after MI. The average NT‐proBNP or apela concentration at 2 and 4 weeks after MI was significantly lower in the apela‐treated group (MI + apela) than in the control group (Figure 1A and B). Correspondingly, the mean value of LVEF at 2 and 4 weeks after MI was significantly higher in the apela‐treated group than in the control group (Figure 1C). The average value of LVEF in apela‐treated mice increased by 32% and 39% at 2‐week and 4‐week post‐MI compared with control mice, respectively. Interestingly, we found a positive correlation between serum levels of apela and NT‐proBNP in the control mice at 2 weeks after MI (*r* = 0.936, *P* < 0.05), whereas there was a negatively correlation between apela (*r* = −0.891, *P* < 0.05) or NT‐proBNP (*r* = −0.976, *P* < 0.05) and LVEF in the control mice at 2‐week post‐MI (Table 1).

**TABLE 1:** Correlations between apela, NT‐proBNP and LVEF in control mice at 2 weeks after MI

	Apela	NT‐proBNP	LVEF (%)
Apela	1	0.936*	−0.891*
NT‐proBNP	0.936*	1	−0.976*
LVEF（%）	−0.891*	‐0.976*	1

Abbreviations: LVEF, Left ventricular ejection fraction, NT‐proBNP, N‐terminal brain natriuretic peptide, MI, Myocardial infarction.

* *P *< 0.05.

### 3.2 Apela improves kidney function in mice after MI

**FIGURE 2:**
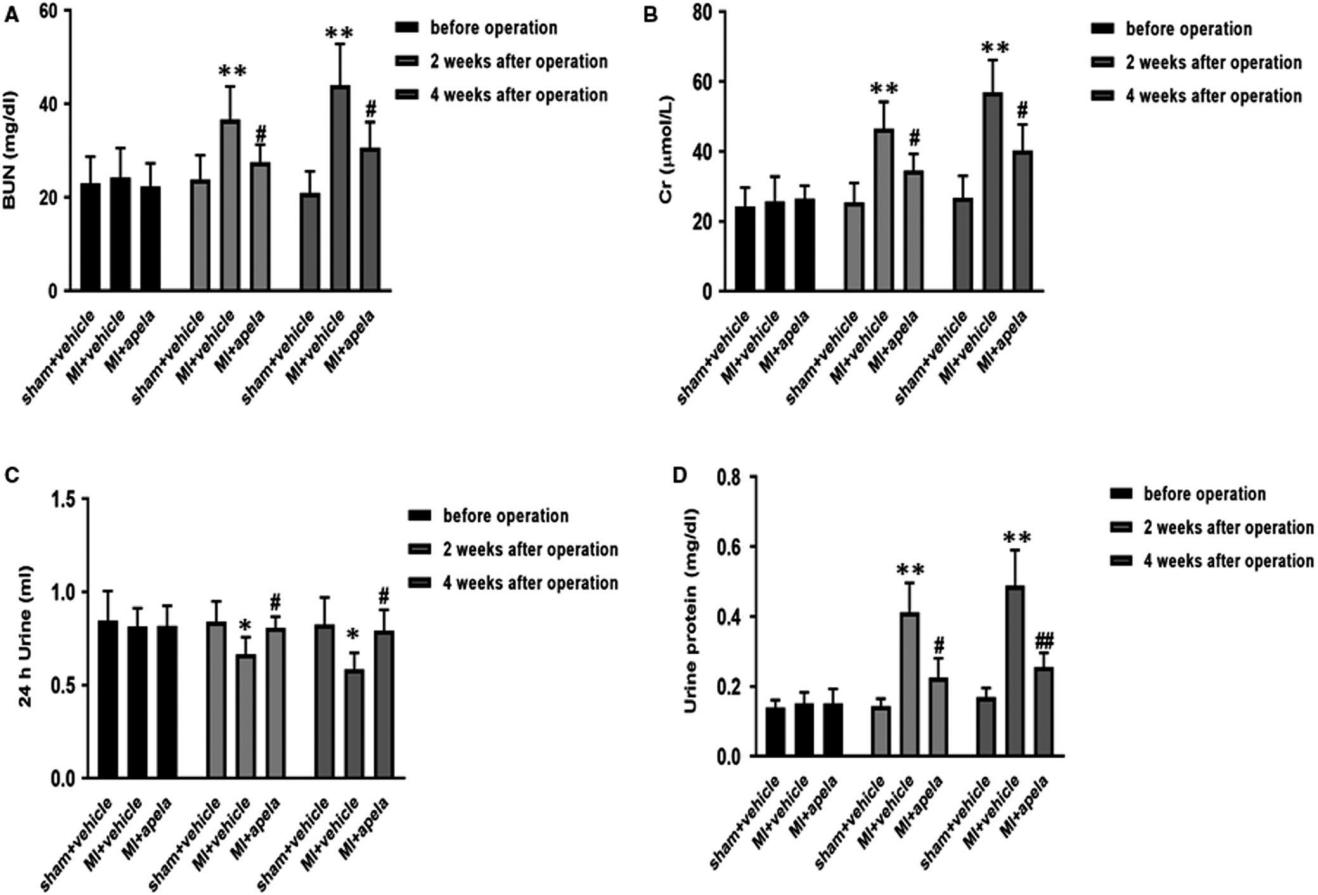
The mice renal function at different times. Serum levels of urea (A) and creatinine (B) in mice. The values of 24‐h urine volume (C) and urine protein content (D) in mice. The results were presented as mean ± SD (n = 5). *P < 0.05, **P < 0.01, vs. sham + vehicle group; ^#^ *P* < 0.05, ^##^ *P* < 0.01, vs. MI + vehicle group

Regarding serum BUN and Cr levels, compared to their baseline levels in mice before MI, their levels were higher at the 2 and 4 weeks after MI. The average BUN and Cr concentrations were lower in apela‐treated mice than in control mice (MI + vehicle) at 2 and 4 weeks after MI (Figure 2A and B). Compared with control mice, the average value of 24‐h urine volume significantly increased at 2 and 4 weeks after MI in apela‐treated mice (Figure 2C); furthermore, in apela‐treated mice, the mean value of 24‐h urine protein content was lower than that in control mice (Figure 2D).

### 3.3 Apela limits MI area and reduces myocardial fibrosis

**FIGURE 3:**
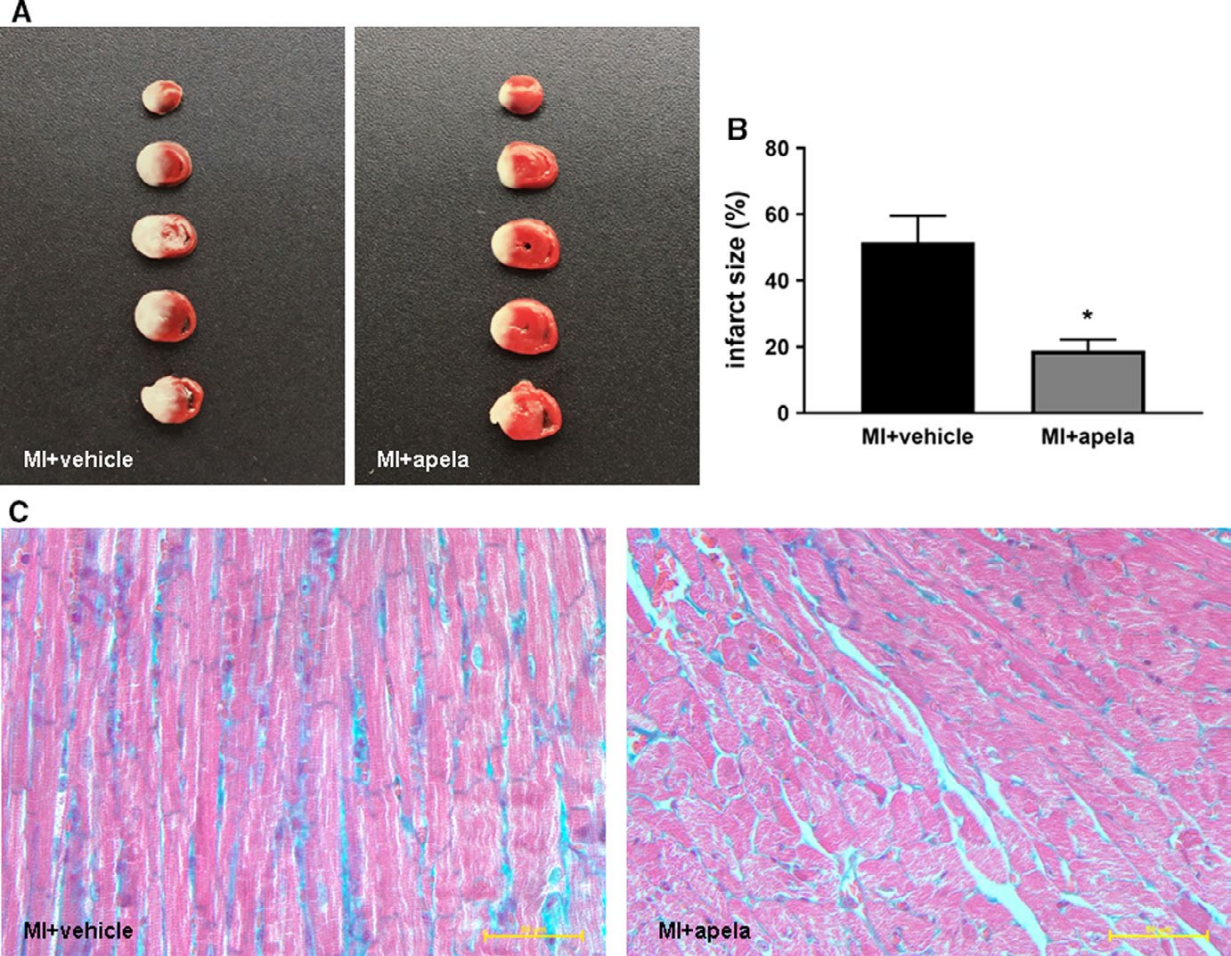
Photographs showing representative TTC staining and histology of heart. The slices were incubated with 2,3,5‐triphenyltetrazolium‐chloride (TTC) for 30 min. Non‐infarcted myocardium was stained brick red, whereas infarcted tissue was unstained (A). The infarct size was measured and calculated as a percentage of the total area (B). Heart histology after Masson's trichrome staining of sections from each group (C).The data are presented as mean ± SD. *P < 0.05, vs. MI + vehicle group

Histopathological results showed that compared with the control group, the apela‐treated group showed significantly decreased mean value of the area of myocardial infarction at 4 weeks after MI (Figure 3A and B). Masson staining of the myocardial tissue in the control group showed obvious interstitial fibrosis at 4 weeks after MI, whereas the myocardial interstitial fibrosis was significantly reduced in apela‐treated mice (Figure 3C).

### 3.4 Apela increases the proportion of CD31‐positive cells in the heart

**FIGURE 4:**
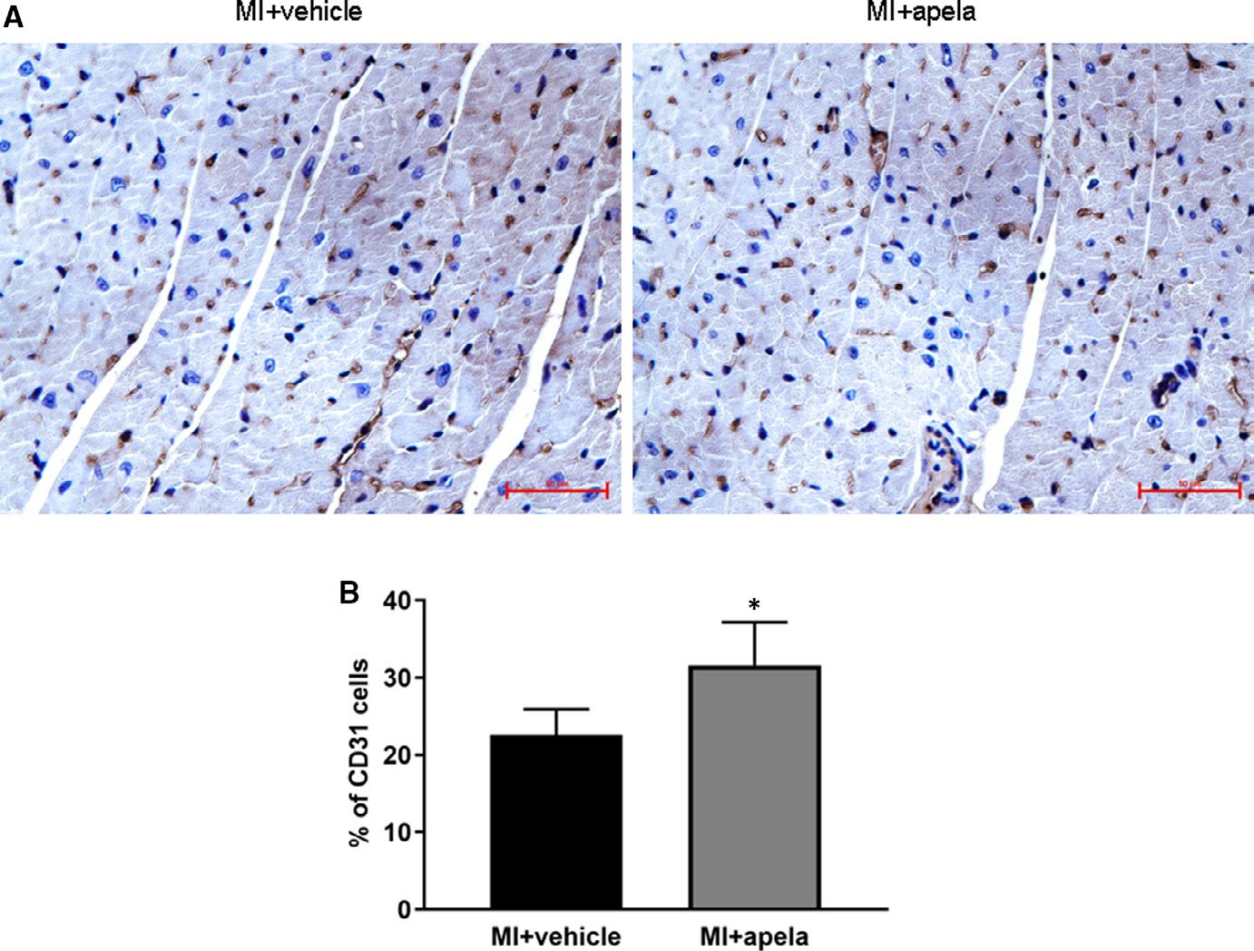
Immunohistochemical (IHC) staining of CD31. Representative photomicrographs of CD31‐positive cells (A). Quantification of CD31‐positive cells in each group (B). The results were presented as mean ± SD. *P < 0.05, vs. MI + vehicle group

To observe the myocardial neovascularization after infarction, we applied immunohistochemistry to detect the expression of neovascular endothelial cell marker CD31.^15^ The results showed that the proportion of CD31‐positive cells near the infarcted area of myocardial tissue was significantly higher in the apela‐treated group than in the control group at 4 weeks after MI (Figure 4A and B).

### 3.5 Apela reduces the proportion of TUNEL‐positive cells in heart and kidney tissues

**FIGURE 5:**
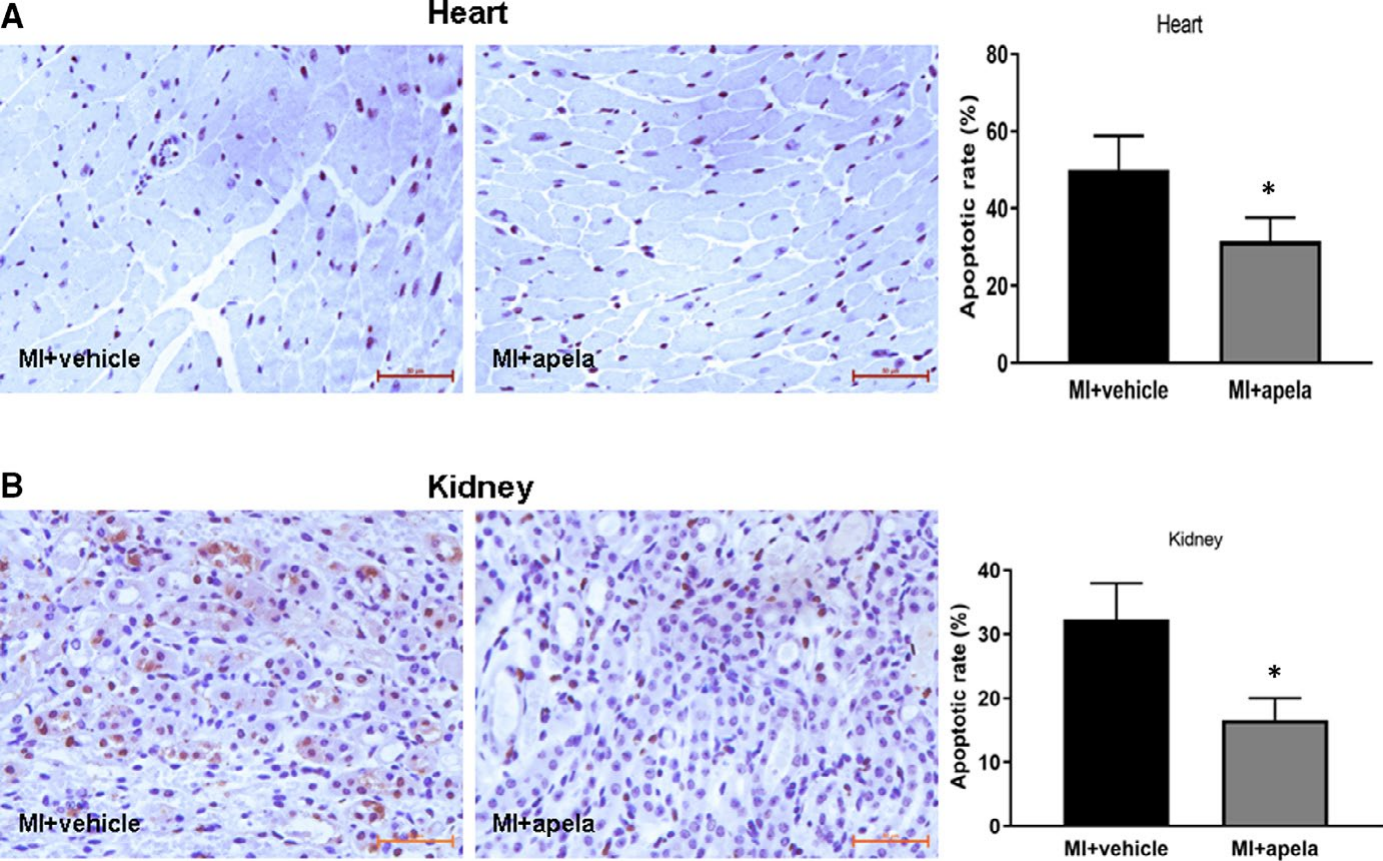
Immunohistochemistry studies of heart and kidney tissue by in situ terminal deoxynucleotidyl transferase‐mediated dUTP nick end‐labelling (TUNEL) reaction. Representative photomicrographs of TUNEL‐positive cells in heart (A) and kidney (B). Quantification of TUNEL‐positive cells in each group (right panel). The results were presented as mean ± SD. *P < 0.05, vs. MI + vehicle group

To observe the apoptosis of heart and kidney tissues after MI, we used TUNEL staining to evaluate the ratio of TUNEL‐positive cells. The results showed that at 4 weeks after MI, the percentage of TUNEL‐positive cells in the non‐infarcted area of the heart was significantly lower in the apela‐treated group than in the control group (Figure 5A). Similarly, the ratio of TUNEL‐positive cells in kidney tissues was significantly lower in apela‐treated mice than in control mice at 4 weeks after MI (Figure 5B).

### 3.6 Apela inhibits MPO expression in kidney tissue

**FIGURE 6:**
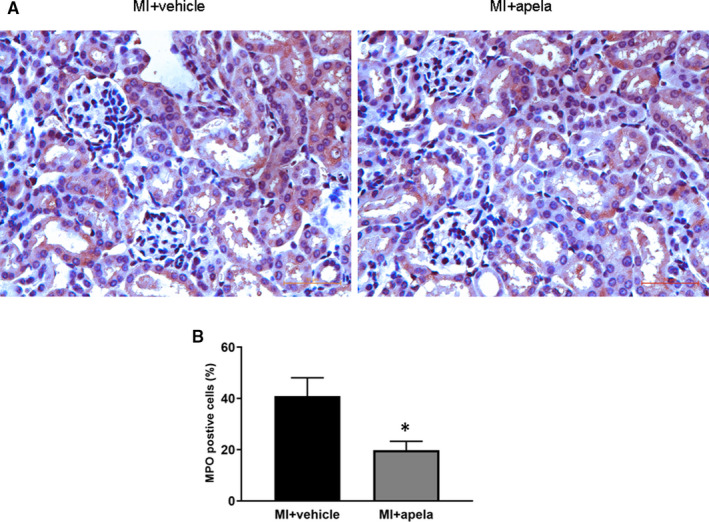
Immunohistochemical (IHC) staining of MPO. Representative photomicrographs of MPO‐positive cells in kidney (A). Quantification of MPO‐positive cells in each group (B). The results were presented as mean ± SD. *P < 0.05, vs. MI + vehicle group

Myeloperoxidase (MPO) is a haemoglobin protein that is rich in neutrophils and is widely used as a marker of inflammation.^16^ Immunohistochemical tests showed that at 4 weeks after MI, the percentage of MPO‐positive cells in kidney tissues of the control group was significantly higher than it was before MI, while the ratio of MPO‐positive cells was significantly lower in the apela‐treated group than in the control group (Figure 6A and B).

## 4 DISCUSSION

The APJ receptor, also known as the orphan G protein‐coupled receptor, was first isolated by O 'Dowd et al. in 1993.^17^ In 1998, Tatemoto et al extracted and purified the first APJ receptor endogenous ligand from bovine gastric secretions and named it apelin.^18^ After 2 decades of research, it has been found that apelin and APJ receptor are widely expressed in mammalian tissues and organs. The apelin/APJ receptor system exerts various biological effects through endocrine or para/autocrine manner, and it interacts with the digestive system, respiratory system, endocrine and reproductive system, central nervous system and cardiovascular system. In particular, apelin plays an important role in the occurrence and development of heart failure.^19^, ^20^

In recent years, two independent research teams from the United States and Singapore have discovered APJ receptor's second endogenous ligand named apela (Elabela/Toddler). The 10 amino acids at the carboxy terminus of the mature 32‐aa‐apela are highly conserved in vertebrates. The molecular structure shows that apela and apelin are basic amino acids and have the same isoelectric point and are APJ receptor's acting ligands; this suggests that apela and apelin have biological similarities.^1^, ^2^ Functional studies have also shown that apela and apelin can promote cardiomyocyte differentiation, induce angiogenesis and dilate aorta in vitro.^21^, ^22^, ^23^, ^24^ In vivo experiments have found that apelin and its structural homologs can alleviate myocardial ischaemia and reperfusion injury.^25^, ^26^

Sato et al. showed that apela can prevent pressure overload heart failure caused by transverse aortic constriction in mice.^27^ Yurdaer et al found that the apela level was positively correlated with NT‐proBNP (*r* = 0.586, *P* < 0.05), whereas the levels of apela ((*r* = −0.551, *P* < 0.05) or NT‐proBNP (*r* = −0.684, *P* < 0.05) were negatively correlated with LVEF in patients with acute MI.^14^ BNP or NT‐proBNP is an important biomarker to evaluate the severity and prognosis of heart function. It is mainly secreted from the ventricle, and its synthesis and secretion are closely related with the ventricular load and wall tension.^28^ As the BNP produced in decompensated heart failure is not enough to antagonize the activated rennin‐angiotensin‐aldosterone system (RAAS) and sympathetic nervous system (SNS), so exogenous recombinant human BNP is widely used to treat decompensated heart failure.^29^ In our study, we also observed that serum NT‐proBNP increased significantly while LVEF declined in mice with AMI. After intervention with exogenous apela, NT‐proBNP decreased significantly and LVEF increased significantly at 2 and 4 weeks after MI. These results suggest that apela can not only prevent heart failure caused by pressure overload but also improve heart failure caused by acute myocardial ischaemia. More interestingly, our results are partially similar to that of Yurdaer et al. We also found that endogenous apela or NT‐proBNP was parallel regulated in mice post‐MI, and the expression of apela was significantly correlated with LVEF at 2 weeks after MI. We speculate that endogenous apela is similar to BNP, which increases in decompensated heart failure, but the amount of endogenous apela is not enough to reverse decreased heart function. Therefore, exogenous apela is needed to improve cardiac function of heart failure. Further large‐sample and perspective clinical studies would help to clarify the relationship between apela levels and heart failure in real world.

Pathological and immunohistological results show that apela can limit the area of myocardial infarction, reduce myocardial interstitial fibrosis and increase the proportion of CD31‐positive cells near the infracted area while significantly reducing the proportion of TUNEL‐positive cells. It is suggested that apela can promote angiogenesis and inhibit cardiomyocyte apoptosis in vivo. We speculate that apela improves cardiac function after MI via the combined effects of limiting infarct size, inhibiting interstitial fibrosis, promoting angiogenesis and reducing myocardial cell apoptosis. Previous studies indicated that about 18.2%–53.3% of C57BL/6 mice died of cardiac rupture within one week after MI.^30^, ^31^ Whether or not apela could prevent cardiac rupture in mice after MI was not examined in the present study, which would be worthwhile to be studied in further research work.

More interestingly, apela can improve not only the heart function in MI mice but also the kidney function. Coquerel et al. found that apela and apelin can improve renal function in septic shock rats and that apela has a stronger ability to inhibit plasma inflammatory cytokines than apelin.^32^ Chen et al. made a mouse model of acute kidney insufficiency (AKI) by renal ischaemia/reperfusion and found that apela can inhibit renal cell apoptosis, DNA damage and inflammation and fibrosis of renal tissue in AKI mice.^33^ In this study, we observed that apela increased 24‐h urine volume and decreased urinary protein excretion significantly in mice after MI. Serum creatinine and urea nitrogen levels were also significantly lower in apela‐treated mice than in control mice. Immunostaining results showed that apela could inhibit the inflammatory response and apoptosis of renal cells in mice after MI.

Acute myocardial infarction weakens myocardial contractility and decreases left ventricular ejection fraction, thus resulting in reduced cardiac output and insufficient perfusion of important organs, such as the kidney. In addition, the activation of the RAAS and the SNS increased the levels of angiotensin II and inflammatory cytokines, causing imbalance in the diastolic and contractile functions of the glomerular afferent and efferent arteries. This eventually leads to a decrease in the glomerular filtration rate.^34^

In recent years, cardiorenal syndrome (CRS) caused by the interconnection and influence of heart and kidney diseases has attracted the attention of cardiologists and nephrologists but mechanistic pathways have yet to be fully elucidated.^35^ Cardiorenal syndrome is a clinical syndrome wherein acute or chronic dysfunction of the heart or kidney causes acute or chronic functional impairment of another organ. The American Heart Association classifies it into five types based on the incidence of heart and kidney disease. Acute renal failure secondary to acute heart failure is called type I cardiorenal syndrome, which is also a common type of cardiorenal syndrome in clinical practice. Once patients with acute heart failure are associated with renal insufficiency, the mortality and disability rates increase significantly. Therefore, effectively preventing cardiorenal syndrome remains a challenge in clinical practice.^36^, ^37^ The data in our study suggest that apela improves cardiac and renal function in mice with acute MI. The most common cause of acute heart failure is AMI, and the abnormal haemodynamics as well as activation of the RAAS and SNS are the major factors that mediate acute heart failure secondary to AMI.^38^ Besides haemodynamic and neurohumoral factors, there are other major pathophysiological mechanisms, including hypertension, inflammation and oxidative stress, involved in CRS.^39^, ^40^ In the present study, the effects of apela on blood pressure, inflammation mediators or other major factors in mice after MI were not examined, which would be worthwhile to be studied in the future.

## 5 CONCLUSIONS

In summary, our results reveal that apela can improve not only cardiac function after MI but also reserve renal function. We suggest that it is possible to develop apela as a clinical drug in the future that can not only treat heart failure but also prevent type I cardiorenal syndrome.

## CONFLICTS OF INTEREST

The authors declare no any conflicts of interest in this work.

## AUTHOR CONTRIBUTION

Yang Pan: Data curation (equal); Formal analysis (equal); Investigation (equal); Methodology (equal); Project administration (equal); Writing‐original draft (equal). Quanyi Li: Data curation (equal); Investigation (equal); Methodology (equal). Hong Yan: Data curation (equal); Methodology (equal). Jin Huang: Investigation (equal); Supervision (equal). Zhi Wang: Data curation (equal); Funding acquisition (lead); Investigation (equal); Methodology (equal); Project administration (lead); Writing‐original draft (equal). Dr. Zhi Wang and Yang Pan designed and performed the study. Yang Pan and Quanyi Li analysed the results. Hong Yan gave us constructive advice in experiment. Dr. Zhi Wang and Yang Pan wrote the manuscript. Dr. Jin Huang reviewed the manuscript. All the authors approved the final version.

## Data Availability

All data generated or analysed during this study are included in this article.
